# Sustained Complete Response to Cytotoxic Therapy and the PARP Inhibitor Veliparib in Metastatic Castration-Resistant Prostate Cancer – A Case Report

**DOI:** 10.3389/fonc.2015.00169

**Published:** 2015-07-22

**Authors:** David J. VanderWeele, Gladell P. Paner, Gini F. Fleming, Russell Z. Szmulewitz

**Affiliations:** ^1^Department of Medicine, University of Chicago, Chicago, IL, USA; ^2^Department of Pathology, University of Chicago, Chicago, IL, USA

**Keywords:** prostate cancer, PARP inhibitor, veliparib, BRCA2, ERG, TP53, complete response

## Abstract

Solid tumors harboring BRCA1 or BRCA2 mutations have been shown to respond to PARP inhibitors. These responses are partial and transient. In this case report, we describe an 82-year-old male with poorly differentiated prostate cancer with metastases to the lung, liver, abdomen, and bowel. Molecular testing demonstrated alterations in BRCA2, ERG, and TP53. Based on this result, he was enrolled in a therapeutic trial and received carboplatin, gemcitabine, and veliparib, to which he had a partial response. He continued to respond while on veliparib maintenance alone, and after 38 cycles he had a sustained complete response. A sustained complete response to PARP inhibitor-based therapy has not previously been described for prostate cancer. This case suggests that cytotoxic therapy in combination with PARP inhibitors may yield exceptional responses, and molecular studies may help guide patient selection for these therapies.

## Introduction

Though genetic alterations serve as a biomarker to guide the use of targeted therapy for many solid tumors, this is rarely employed for prostate cancer. Given the important role of the androgen receptor (AR) in prostate cancer (1), AR-targeted therapies are the backbone of therapy for almost all patients and no specific genetic test is warranted. Emerging data suggest, however, that for some patients specific genetic derangements may predict response to additional therapies. Preclinical data indicate that tumors that harbor an ETS family rearrangement, such as TMPRSS2-ERG, the most frequent cancer rearrangement, may be responsive to PARP inhibitors (2). Those with a neuroendocrine phenotype typically harbor amplification or overexpression of AURKA or MYCN and are hypothesized to respond to an AURKA inhibitor (3). Clinical trials are underway to evaluate the use of PARP inhibitors (NCT01576172) and AURKA inhibitors (NCT01799278) based on genetic features.

Men who are carriers of BRCA2 mutations are at higher risk of developing prostate cancer than non-carriers, and when they are diagnosed with prostate cancer, it tends to be with higher grade, more aggressive disease (4–6). Evidence from prostate cancer and other tumor types has shown efficacy of PARP inhibitors for tumors with BRCA1 or BRCA2 alterations (7–9), but without complete responses. Preclinical data from the TP53-altered prostate cancer line PC3 have demonstrated synergy between veliparib and cytotoxic therapies (10), and those with alterations in ATM, another DNA-damage response gene, have also been reported to respond to PARP inhibitors (11).

We present here a patient with an aggressive prostate cancer with visceral metastases harboring a BRCA2 deletion and TMPRSS2-ERG rearrangement, along with TP53 alteration. Based on his BRCA2 deletion, he was enrolled in a clinical trial and treated with carboplatin, gemcitabine, and the PARP inhibitor veliparib. He had a complete response to therapy, and continues on veliparib maintenance therapy 30 months after enrolling in the trial, with no evidence of disease. To our knowledge, this is the first report of complete response to PARP inhibitor-based therapy for a patient with prostate cancer.

## Case Report

The patient, an 82-year-old man, underwent a surgical procedure at age 61 for treatment of urinary retention due to benign prostatic hypertrophy. Twelve years later, he developed similar symptoms, and a prostate biopsy was performed, showing conventional adenocarcinoma of the prostate, Gleason score 4 + 3 = 7. He underwent external beam radiation therapy, and his PSA nadired at 0.5 ng/ml but rose to 13.5 ng/ml 3 years after radiation therapy. He began androgen deprivation therapy (ADT) with a luteinizing hormone-releasing hormone analog. Despite this, over the next few years he had multiple surgical procedures for continued urinary retention.

**Figure 1:**
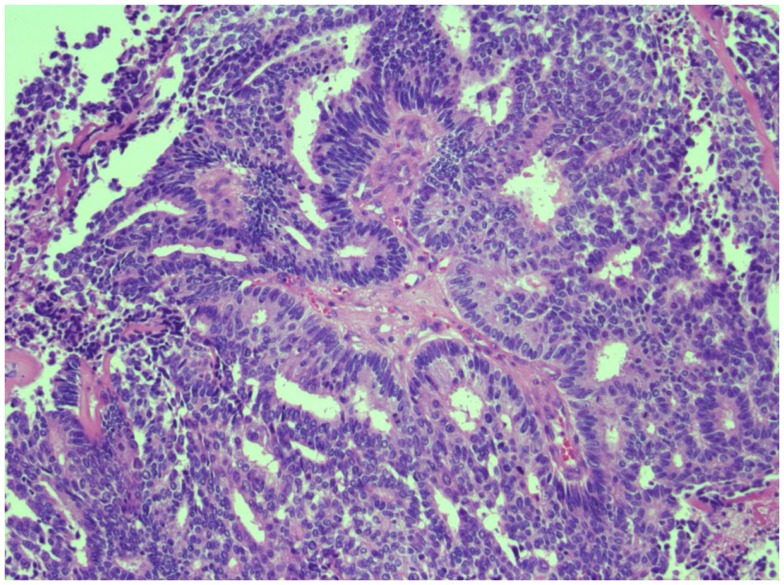
**H & E stain of poorly differentiated prostate cancer**. Representative image of high-grade prostate carcinoma from the cystoprostatectomy specimen exhibiting cribriform pattern and cell necrosis.

A transurethral resection of the prostate performed 6 years after radiation therapy showed sarcomatoid prostatic adenocarcinoma involving the prostatic urethra and bladder neck. Due to persistent hematuria and lower urinary tract symptoms, the patient underwent a salvage cystoprostatectomy with ileal conduit and descending loop colostomy. Pathology revealed high-grade prostatic adenocarcinoma with extensive sarcomatoid and focal neuroendocrine differentiation diffusely involving the prostate and extending into the seminal vesicles, urinary bladder wall including mucosa and large bowel wall, pT4N0 with 20 regional lymph nodes negative for malignancy (Figure 1). His PSA at the time of cystoprostatectomy was <0.05 ng/ml.

**Figure 2:**
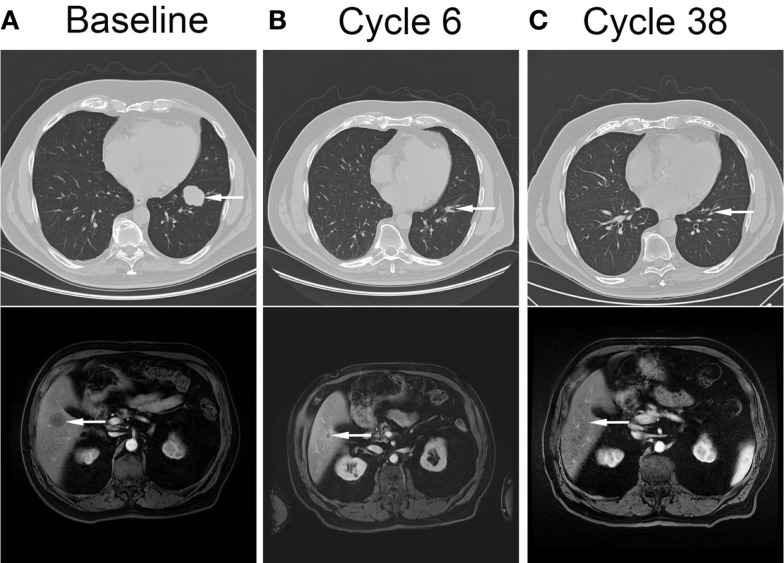
**Complete resolution of metastatic disease in response to cytotoxic therapy and veliparib**. Computed tomography images showing pulmonary (top panel, arrow) and liver (bottom panel, arrow) metastases at baseline **(A)**, after completion of cytotoxic therapy and veliparib **(B)**, and after 38 cycles of veliparib maintenance therapy **(C)**.

The patient continued on ADT following cystoprostatectomy but developed recurrence of his disease, with radiographically detected masses within the pelvis and lungs 10 months after his surgery. His PSA remained undetectable. An endoscopic biopsy of a colonic mass showed poorly differentiated carcinoma invading colonic mucosa. The tumor cells exhibited similar morphologic features to those in the cystoprostatectomy specimen. He remained minimally symptomatic and was managed conservatively. Six months after his recurrence, he developed worsening rectal bleeding. Sigmoidoscopy showed progression of his metastatic disease in his rectum. Imaging confirmed bowel wall thickening and showed other pelvic disease; progression of his lung metastases, largest measuring 3.5 cm; and new liver metastases, the largest 3.1 × 3.0 cm (Figure 2A).

Tumor tissue from the patient’s cystoprostatectomy was sent to Foundation One for genetic testing. Three genomic alterations were identified: BRCA2 loss, TMPRSS2-ERG fusion, and TP53 F270V. The BRCA2 loss and TMPRSS2-ERG translocation both suggested that he may have increased sensitivity to a PARP inhibitor, and he provided consent for and was enrolled in an Internal Review Board-approved phase I clinical trial for patients with documented BRCA1/BRCA2 mutations (NCT01063816, IRB 09267) consisting of six 21-day cycles of carboplatin AUC = 4 day 1, gemcitabine 800 mg/m^2^ days 1 and 8, and veliparib 250 mg PO BID daily, to be followed by veliparib 250 mg PO BID maintenance therapy. The patient’s course was complicated by diarrhea, thrombocytopenia, anemia requiring transfusion, and fatigue. Veliparib was held in cycle 2 for thrombocytopenia, and restarted with cycle 3 at 210 mg PO BID. Gemcitabine was reduced to 400 mg/m^2^ on cycle 3 day 8.

Restaging radiographic imaging was performed after three cycles of therapy, which showed a partial response within the liver and lungs. The rectal bleeding had resolved at this time. The patient completed three additional cycles of therapy, dose reduced due to thrombocytopenia and consisting of carboplatin AUC = 4 day 1, gemcitabine 400 mg/m^2^ day 1, and veliparib 210 mg PO BID. Thrombocytopenia and diarrhea resolved, and anemia and fatigue improved. Repeat staging at the completion of six cycles showed continued decrease in size of lung metastases, stable liver metastases, and resolution of bowel wall thickening (Figure 2B). He started maintenance therapy with veliparib 210 mg PO BID and continued to have improvement in his metastases. Ten months after completion of therapy, he had complete resolution of all sites of metastatic disease, and after 38 cycles of therapy continues to have no evidence of disease (Figure 2C). Currently he continues on veliparib therapy with minimal toxicity.

## Discussion

One could consider castration the first molecularly targeted therapy in oncology, reducing activity of AR, which drives prostate cancer growth and proliferation. Since castration was described as effective therapy for prostate cancer seven decades ago, few therapies for prostate cancer have found targets beyond AR. That is beginning to change, however, with therapies targeting PARP and AURKA now in clinical trials for the disease. As the development of these therapies progresses, we need ways to identify which patients will most benefit from these therapies.

We describe here a complete response to veliparib-containing therapy in a patient with BRCA2 deletion, TMPRSS2-ERG translocation, and TP53 mutation. Preclinical and clinical evidence suggest that all three alterations may contribute to the patient’s dramatic response to therapy.

**Figure 3:**
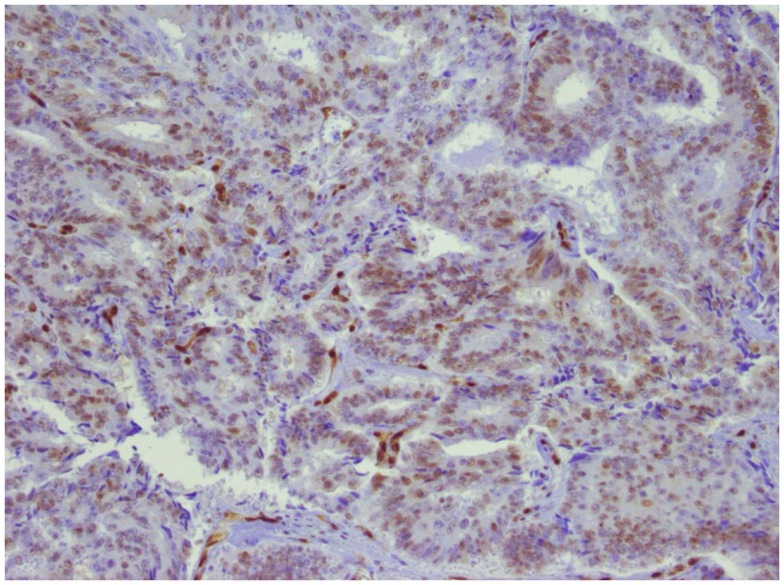
**ERG expression in prostate tumor**. Immunohistochemical analysis of the primary tumor. ERG expression is indicated by brown chromogen in the nucleus. Blue represents hematoxylin counterstain.

Sensitivity of tumors harboring ERG translocations to PARP inhibitors appears to be dependent on expression of *ERG* and interaction between *ERG* and *DNAPK*. Hormone naïve cancers harboring ERG rearrangements typically express *ERG* protein, whereas those lacking ERG rearrangements do not. For those with lethal, castrate-resistant disease, however, *ERG* expression is variable despite the presence of ERG translocation (12). Since expression of the PSA gene KLK3 is regulated by AR, as is TMPRSS2, the patient’s low PSA value suggested that *ERG* might not be expressed. *ERG* expression was confirmed, however, with immunohistochemical analysis (Figure 3).

TMPRSS2-ERG is the most frequent genomic alteration described in localized prostate cancer, with 40–50% of patients harboring this translocation, and others harboring rearrangements involving other ETS family members (13, 14). BRCA2 alterations are less common, but those with germline BRCA2 alterations are known to be at high risk of prostate cancer diagnosis and high risk of having aggressive disease. It is likely that those with aggressive disease are more likely to harbor these particular alterations. Indeed, in cases of localized prostate cancer sequenced by The Cancer Genome Atlas project, the percent of patients with ERG fusions, BRCA2 deletion or mutation, or both is 35, 4, and 2%, respectively. A recent report on castrate-resistant prostate cancer found that 7% of cases have missense or truncating mutations in BRCA2, and an additional 12% have alterations in other DNA repair/recombination genes associated with response to PARP inhibitors (15). In cases of end-stage prostate cancer sequenced by the University of Michigan (16), the percent of patients with ERG fusions, BRCA2 deletion or mutation, or both is 48, 13, and 8%, respectively. Of those harboring both BRCA2 and ERG alterations, 40% also harbor a deletion in TP53. Though the percentages are small, given the large number of patients with metastatic prostate cancer, this may represent a large cohort of patients who could be considered for therapy similar to what our patient received. It is possible that alterations in other DNA-damage pathway genes in addition to TP53 would sensitize to PARP inhibitors. The hypothesis that patients with similar known genetic alterations will benefit from a similar treatment regimen needs to be tested in the setting of a prospective clinical trial.

It is unknown if all the components of this therapeutic regimen were necessary in order for this patient to achieve his therapeutic success, as he received carboplatin and gemcitabine in addition to veliparib. However, his disease continued to respond to veliparib alone without achieving a complete response until 10 months after completion of cytotoxic chemotherapy, supporting the notion that there was additional benefit to the veliparib. Although it is conceivable that the patient could have responded to the PARP inhibitor alone (8), other data suggest synergy with cytotoxic therapy (17–21). The optimal duration of treatment is also unknown in this case. The patient has had a sustained complete response 32 cycles after completion of chemotherapy, on veliparib alone. It is unknown if discontinuing veliparib would result in disease recurrence or continued durable response.

Intentionally designed, targeted therapies offer much hope for the treatment of advanced malignancy but often fail to meet expectations. Better combinations of therapy, better biomarkers, or both are needed to help guide therapy decisions. Exploration of the molecular characteristics of those with exceptional responses to therapy is an important tool in improving the use of the therapies we have at hand.

## Conflict of Interest Statement

The authors declare that the research was conducted in the absence of any commercial or financial relationships that could be construed as a potential conflict of interest.
